# Dichlorido(2,9-diprop­oxy-1,10-phenanthroline-κ^2^ *N*,*N*′)cadmium(II)

**DOI:** 10.1107/S1600536809038124

**Published:** 2009-09-26

**Authors:** Cao-Yuan Niu, Xian-Fu Zheng, Yu-Li Dang

**Affiliations:** a College of Sciences, Henan Agricultural University, Zhengzhou 450002, People’s Republic of China

## Abstract

In the title complex, [CdCl_2_(C_18_H_20_N_2_O_2_)], the Cd^II^ ion is coordinated by two N atoms from a bis-chelating 2,9-diprop­oxy-1,10-phenanthroline ligand and two Cl atoms in a distorted tetra­hedral environment. The two Cd—Cl bond distances are significantly different from each other and the N—Cd—N bond angle is acute. In the crystal structure, there are π–π stacking inter­actions between symmetry-related phenanthroline ring systems, with a centroid–centroid distance of 3.585 (3) Å.

## Related literature

For details of the coordination chemistry of 1,10-phenanthroline derivatives, see: Arpi *et al.* (2006 ▶); Bie *et al.* (2006 ▶). For synthetic details, see: Pijper *et al.* (1984 ▶).
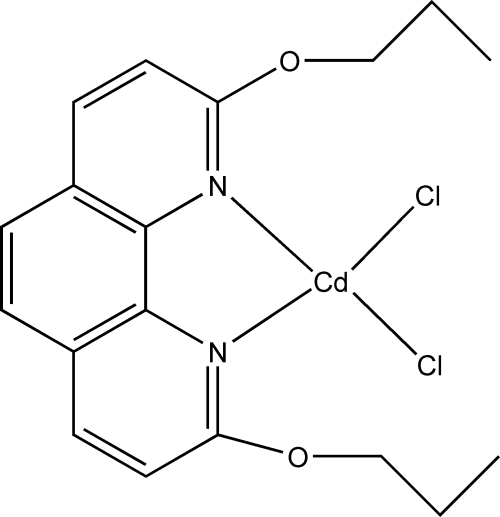


## Experimental

### 

#### Crystal data

[CdCl_2_(C_18_H_20_N_2_O_2_)]
*M* *_r_* = 479.66
Tetragonal, 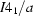

*a* = 31.3159 (10) Å
*c* = 8.1662 (5) Å
*V* = 8008.5 (6) Å^3^
*Z* = 16
Mo *K*α radiation
μ = 1.37 mm^−1^
*T* = 291 K
0.18 × 0.07 × 0.04 mm

#### Data collection

Bruker APEXII CCD diffractometer
Absorption correction: multi-scan (*SADABS*; Bruker, 2005 ▶) *T*_min_ = 0.791, *T*_max_ = 0.947
21000 measured reflections
3722 independent reflections
2656 reflections with *I* > 2σ(*I*)
*R*_int_ = 0.051

#### Refinement

*R*[*F* ^2^ > 2σ(*F* ^2^)] = 0.039
*wR*(*F* ^2^) = 0.101
*S* = 1.02
3722 reflections
228 parameters
21 restraints
H-atom parameters constrained
Δρ_max_ = 0.80 e Å^−3^
Δρ_min_ = −0.41 e Å^−3^

### 

Data collection: *APEX2* (Bruker, 2005 ▶); cell refinement: *SAINT* (Bruker, 2005 ▶); data reduction: *SAINT*; program(s) used to solve structure: *SHELXS97* (Sheldrick, 2008 ▶); program(s) used to refine structure: *SHELXL97* (Sheldrick, 2008 ▶); molecular graphics: *DIAMOND* (Brandenburg, 2005 ▶); software used to prepare material for publication: *SHELXL97*.

## Supplementary Material

Crystal structure: contains datablocks I, global. DOI: 10.1107/S1600536809038124/lh2892sup1.cif

Structure factors: contains datablocks I. DOI: 10.1107/S1600536809038124/lh2892Isup2.hkl

Additional supplementary materials:  crystallographic information; 3D view; checkCIF report

## Figures and Tables

**Table:** 

Cd1—N2	2.285 (4)
Cd1—N1	2.297 (4)
Cd1—Cl2	2.3623 (14)
Cd1—Cl1	2.4182 (15)

**Table:** 

N2—Cd1—N1	72.66 (13)
N2—Cd1—Cl2	121.73 (11)
N1—Cd1—Cl2	119.18 (10)
N2—Cd1—Cl1	103.88 (10)
N1—Cd1—Cl1	109.63 (9)
Cl2—Cd1—Cl1	120.03 (5)

**Fig. 1.**
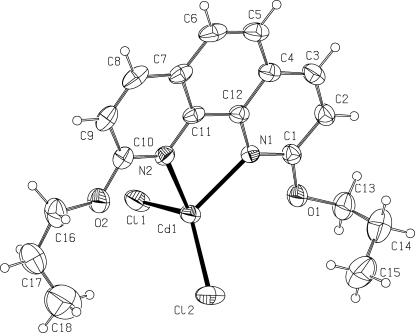
The molecular structure of the title compound, showing the atom-labelling scheme. Displacement ellipsoids are drawn at the 30% probability level and H atoms are shown as small spheres of arbitrary radii.

**Fig. 2.**
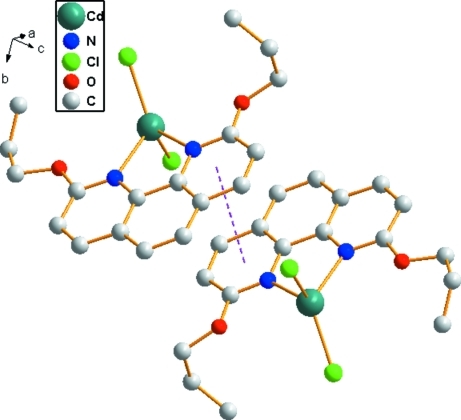
Packing diagram showing the π-π interaction (purple dotted line). All H atoms have been omitted for clarity.

## supplementary crystallographic information

### Comment

The compound 1,10-phenanthroline and its derivatives have been used with d^10^ metals to synthesize some luminescent materials (Arpi, *et al.*, 2006; Bie, *et al.*, 2006). The compound 2,9-diethoxy-1,10-phenanthroline has been shown to possesses antimycoplasmal activity in the presence of copper (Pijper, *et al.*, 1984). Herein, we present the crystal structure of the title compound.

The molecular structure of the title compound is shown in Fig.1. The Cd^II^ ion is four-coordinated to two nitrogen atoms from one 1,10-phenanthroline ring (N1, N2) and two chlorine atoms (Cl1, Cl2), defining a distorted tetrahedral coordination geometry. The two Cd—Cl bond distances are significantly different from each other. The the N—Zn—N bond angle is acute. In the crystal structure, there are π–π stacking interactions between phenanthroline ring systems with centroid to centroid distances of 3.5847 (1) Å (Fig. 2).

### Experimental

2,9-Dipropoxy-1,10-phenanthroline was prepared according to the literature procedure (Pijper, *et al.*, 1984). Slow evaporation of a mixture of the ligand (0.027 g, 0.1 mmol) and cadmium dichloride (0.016 g, 0.1 mmol) in 30 ml methanol afforded suitable colourless block crystals in about 7 days (yield about 45%).

### Refinement

C-bound H atoms were placed in calculated positions and refined using a riding model [C—H = 0.93 Å and *U*_iso_(H) = 1.2*U*_eq_(C) for aromatic H atoms, C—H = 0.97 Å and *U*_iso_(H) = 1.2*U*_eq_(C) for methylene H atoms and C—H = 0.96 Å and *U*_iso_(H) = 1.5*U*_eq_(C) for methyl H atoms]. The final difference Fourier map had a highest peak 0.94 Å from atom C17 and a deepest hole at 0.85 Å from atom H18, but were otherwise featureless.

### Crystal data

**Table:**

[CdCl_2_(C_18_H_20_N_2_O_2_)]	*D*_x_ = 1.591 Mg m^−^^3^
*M**_r_* = 479.66	Mo *K*α radiation, λ = 0.71073 Å
Tetragonal, *I*4_1_/*a*	Cell parameters from 3326 reflections
Hall symbol: -I 4ad	θ = 2.6–25.5°
*a* = 31.3159 (10) Å	µ = 1.37 mm^−^^1^
*c* = 8.1662 (5) Å	*T* = 291 K
*V* = 8008.5 (6) Å^3^	Needle, colourless
*Z* = 16	0.18 × 0.07 × 0.04 mm
*F*(000) = 3840	

### Data collection

**Table:**

Bruker APEXII CCD diffractometer	3722 independent reflections
Radiation source: fine-focus sealed tube	2656 reflections with *I* > 2σ(*I*)
graphite	*R*_int_ = 0.051
φ and ω scans	θ_max_ = 25.5°, θ_min_ = 2.6°
Absorption correction: multi-scan (*SADABS*; Bruker, 2005)	*h* = −36→37
*T*_min_ = 0.791, *T*_max_ = 0.947	*k* = −37→29
21000 measured reflections	*l* = −8→9

### Refinement

**Table:**

Refinement on *F*^2^	Primary atom site location: structure-invariant direct methods
Least-squares matrix: full	Secondary atom site location: difference Fourier map
*R*[*F*^2^ > 2σ(*F*^2^)] = 0.039	Hydrogen site location: inferred from neighbouring sites
*wR*(*F*^2^) = 0.101	H-atom parameters constrained
*S* = 1.01	*w* = 1/[σ^2^(*F*_o_^2^) + (0.0421*P*)^2^ + 20.3768*P*] where *P* = (*F*_o_^2^ + 2*F*_c_^2^)/3
3722 reflections	(Δ/σ)_max_ = 0.001
228 parameters	Δρ_max_ = 0.80 e Å^−^^3^
21 restraints	Δρ_min_ = −0.41 e Å^−^^3^

### Special details

**Table:**

Geometry. All e.s.d.'s (except the e.s.d. in the dihedral angle between two l.s. planes) are estimated using the full covariance matrix. The cell e.s.d.'s are taken into account individually in the estimation of e.s.d.'s in distances, angles and torsion angles; correlations between e.s.d.'s in cell parameters are only used when they are defined by crystal symmetry. An approximate (isotropic) treatment of cell e.s.d.'s is used for estimating e.s.d.'s involving l.s. planes.
Refinement. Refinement of *F*^2^ against ALL reflections. The weighted *R*-factor *wR* and goodness of fit *S* are based on *F*^2^, conventional *R*-factors *R* are based on *F*, with *F* set to zero for negative *F*^2^. The threshold expression of *F*^2^ > σ(*F*^2^) is used only for calculating *R*-factors(gt) *etc*. and is not relevant to the choice of reflections for refinement. *R*-factors based on *F*^2^ are statistically about twice as large as those based on *F*, and *R*- factors based on ALL data will be even larger.

### Fractional atomic coordinates and isotropic or equivalent isotropic displacement parameters (Å^2^)

**Table:**

	*x*	*y*	*z*	*U*_iso_*/*U*_eq_	
Cd1	0.329113 (11)	0.831007 (11)	0.02856 (5)	0.05269 (15)	
N1	0.26377 (11)	0.80406 (11)	−0.0466 (4)	0.0434 (9)	
N2	0.32414 (12)	0.76370 (11)	0.1350 (5)	0.0498 (9)	
Cl1	0.37894 (5)	0.82330 (5)	−0.19572 (19)	0.0758 (4)	
Cl2	0.33196 (5)	0.89205 (5)	0.19815 (19)	0.0805 (5)	
O1	0.24631 (11)	0.86473 (11)	−0.1694 (4)	0.0632 (9)	
O2	0.38663 (13)	0.77153 (12)	0.2600 (5)	0.0803 (12)	
C1	0.23473 (14)	0.82433 (15)	−0.1329 (6)	0.0497 (11)	
C2	0.19693 (16)	0.80477 (18)	−0.1842 (6)	0.0606 (13)	
H2	0.1768	0.8200	−0.2440	0.073*	
C3	0.19006 (16)	0.76326 (19)	−0.1456 (6)	0.0640 (14)	
H3	0.1653	0.7498	−0.1815	0.077*	
C4	0.22027 (16)	0.73999 (15)	−0.0509 (6)	0.0534 (12)	
C5	0.21500 (19)	0.69696 (18)	−0.0030 (7)	0.0678 (16)	
H5	0.1904	0.6823	−0.0328	0.081*	
C6	0.2456 (2)	0.67679 (17)	0.0860 (8)	0.0715 (16)	
H6	0.2417	0.6484	0.1154	0.086*	
C7	0.28325 (17)	0.69825 (15)	0.1348 (6)	0.0564 (13)	
C8	0.3164 (2)	0.67952 (17)	0.2272 (7)	0.0697 (16)	
H8	0.3142	0.6510	0.2572	0.084*	
C9	0.3512 (2)	0.70209 (17)	0.2734 (7)	0.0659 (15)	
H9	0.3727	0.6895	0.3355	0.079*	
C10	0.35403 (17)	0.74526 (16)	0.2245 (6)	0.0582 (13)	
C11	0.28890 (15)	0.74128 (14)	0.0899 (6)	0.0474 (11)	
C12	0.25695 (14)	0.76273 (14)	−0.0050 (5)	0.0464 (11)	
C13	0.2158 (2)	0.8939 (2)	−0.2393 (8)	0.0847 (19)	
H13A	0.1992	0.8794	−0.3228	0.102*	
H13B	0.2307	0.9176	−0.2903	0.102*	
C14	0.1869 (3)	0.9102 (3)	−0.1088 (12)	0.118 (3)	
H14A	0.1706	0.8865	−0.0652	0.141*	
H14B	0.1670	0.9302	−0.1572	0.141*	
C15	0.2103 (3)	0.9323 (3)	0.0324 (12)	0.133 (3)	
H15A	0.2297	0.9125	0.0826	0.199*	
H15B	0.1899	0.9418	0.1121	0.199*	
H15C	0.2259	0.9564	−0.0089	0.199*	
C16	0.41947 (19)	0.7591 (2)	0.3744 (9)	0.094 (2)	
H16A	0.4306	0.7311	0.3465	0.112*	
H16B	0.4081	0.7580	0.4848	0.112*	
C17	0.4545 (3)	0.7927 (3)	0.3621 (14)	0.155 (4)	
H17A	0.4765	0.7853	0.4407	0.187*	
H17B	0.4671	0.7903	0.2541	0.187*	
C18	0.4437 (3)	0.8368 (3)	0.3878 (16)	0.182 (4)	
H18A	0.4241	0.8459	0.3045	0.272*	
H18B	0.4691	0.8538	0.3825	0.272*	
H18C	0.4307	0.8400	0.4935	0.272*	

### Atomic displacement parameters (Å^2^)

**Table:**

	*U*^11^	*U*^22^	*U*^33^	*U*^12^	*U*^13^	*U*^23^
Cd1	0.0529 (2)	0.0449 (2)	0.0603 (3)	−0.00643 (15)	−0.00647 (17)	−0.00164 (17)
N1	0.044 (2)	0.041 (2)	0.045 (2)	0.0000 (16)	−0.0002 (17)	−0.0051 (17)
N2	0.056 (2)	0.042 (2)	0.052 (2)	0.0049 (18)	−0.0024 (19)	−0.0005 (18)
Cl1	0.0642 (8)	0.0880 (10)	0.0751 (10)	−0.0247 (7)	0.0081 (7)	−0.0212 (8)
Cl2	0.0959 (11)	0.0639 (9)	0.0818 (10)	−0.0271 (8)	0.0134 (8)	−0.0216 (8)
O1	0.059 (2)	0.060 (2)	0.071 (2)	0.0051 (17)	−0.0177 (18)	0.0077 (18)
O2	0.073 (3)	0.070 (3)	0.098 (3)	0.009 (2)	−0.033 (2)	0.009 (2)
C1	0.046 (3)	0.058 (3)	0.045 (3)	0.001 (2)	−0.003 (2)	−0.005 (2)
C2	0.051 (3)	0.074 (4)	0.057 (3)	0.001 (3)	−0.009 (2)	−0.010 (3)
C3	0.050 (3)	0.084 (4)	0.058 (3)	−0.015 (3)	0.005 (3)	−0.022 (3)
C4	0.056 (3)	0.055 (3)	0.049 (3)	−0.010 (2)	0.013 (2)	−0.011 (2)
C5	0.071 (4)	0.060 (3)	0.073 (4)	−0.022 (3)	0.023 (3)	−0.018 (3)
C6	0.094 (4)	0.043 (3)	0.078 (4)	−0.018 (3)	0.033 (4)	−0.011 (3)
C7	0.075 (3)	0.041 (3)	0.053 (3)	0.000 (2)	0.021 (3)	−0.004 (2)
C8	0.100 (5)	0.042 (3)	0.067 (4)	0.012 (3)	0.029 (3)	0.005 (3)
C9	0.082 (4)	0.056 (3)	0.060 (3)	0.024 (3)	0.008 (3)	0.010 (3)
C10	0.061 (3)	0.055 (3)	0.058 (3)	0.010 (2)	0.001 (3)	0.002 (3)
C11	0.061 (3)	0.037 (2)	0.044 (3)	−0.004 (2)	0.015 (2)	−0.006 (2)
C12	0.049 (3)	0.046 (3)	0.045 (3)	−0.005 (2)	0.009 (2)	−0.008 (2)
C13	0.080 (4)	0.080 (4)	0.094 (5)	0.018 (3)	−0.021 (4)	0.017 (4)
C14	0.101 (6)	0.116 (6)	0.136 (7)	0.036 (5)	0.000 (6)	0.013 (6)
C15	0.142 (8)	0.119 (7)	0.138 (8)	0.032 (6)	0.017 (6)	−0.025 (6)
C16	0.076 (4)	0.101 (5)	0.104 (5)	0.015 (4)	−0.034 (4)	0.017 (4)
C17	0.111 (6)	0.151 (6)	0.204 (8)	0.025 (5)	−0.095 (6)	−0.038 (6)
C18	0.170 (8)	0.168 (7)	0.207 (8)	−0.013 (6)	−0.038 (7)	−0.011 (7)

### Geometric parameters (Å, °)

**Table:**

Cd1—N2	2.285 (4)	C7—C8	1.411 (8)
Cd1—N1	2.297 (4)	C8—C9	1.353 (8)
Cd1—Cl2	2.3623 (14)	C8—H8	0.9300
Cd1—Cl1	2.4182 (15)	C9—C10	1.412 (7)
N1—C1	1.314 (5)	C9—H9	0.9300
N1—C12	1.355 (5)	C11—C12	1.433 (6)
N2—C10	1.321 (6)	C13—C14	1.488 (10)
N2—C11	1.359 (6)	C13—H13A	0.9700
O1—C1	1.349 (6)	C13—H13B	0.9700
O1—C13	1.439 (6)	C14—C15	1.530 (11)
O2—C10	1.343 (6)	C14—H14A	0.9700
O2—C16	1.443 (6)	C14—H14B	0.9700
C1—C2	1.397 (7)	C15—H15A	0.9600
C2—C3	1.355 (7)	C15—H15B	0.9600
C2—H2	0.9300	C15—H15C	0.9600
C3—C4	1.423 (7)	C16—C17	1.524 (8)
C3—H3	0.9300	C16—H16A	0.9700
C4—C12	1.403 (6)	C16—H16B	0.9700
C4—C5	1.413 (7)	C17—C18	1.436 (8)
C5—C6	1.358 (8)	C17—H17A	0.9700
C5—H5	0.9300	C17—H17B	0.9700
C6—C7	1.415 (7)	C18—H18A	0.9600
C6—H6	0.9300	C18—H18B	0.9600
C7—C11	1.408 (6)	C18—H18C	0.9600
			
N2—Cd1—N1	72.66 (13)	O2—C10—C9	125.0 (5)
N2—Cd1—Cl2	121.73 (11)	N2—C11—C7	121.7 (5)
N1—Cd1—Cl2	119.18 (10)	N2—C11—C12	118.1 (4)
N2—Cd1—Cl1	103.88 (10)	C7—C11—C12	120.1 (4)
N1—Cd1—Cl1	109.63 (9)	N1—C12—C4	123.1 (4)
Cl2—Cd1—Cl1	120.03 (5)	N1—C12—C11	118.3 (4)
C1—N1—C12	119.1 (4)	C4—C12—C11	118.6 (4)
C1—N1—Cd1	125.6 (3)	O1—C13—C14	109.7 (5)
C12—N1—Cd1	115.1 (3)	O1—C13—H13A	109.7
C10—N2—C11	120.0 (4)	C14—C13—H13A	109.7
C10—N2—Cd1	124.4 (3)	O1—C13—H13B	109.7
C11—N2—Cd1	115.4 (3)	C14—C13—H13B	109.7
C1—O1—C13	120.3 (4)	H13A—C13—H13B	108.2
C10—O2—C16	121.0 (4)	C13—C14—C15	113.8 (7)
N1—C1—O1	112.7 (4)	C13—C14—H14A	108.8
N1—C1—C2	122.4 (5)	C15—C14—H14A	108.8
O1—C1—C2	124.9 (5)	C13—C14—H14B	108.8
C3—C2—C1	119.0 (5)	C15—C14—H14B	108.8
C3—C2—H2	120.5	H14A—C14—H14B	107.7
C1—C2—H2	120.5	C14—C15—H15A	109.5
C2—C3—C4	120.8 (5)	C14—C15—H15B	109.5
C2—C3—H3	119.6	H15A—C15—H15B	109.5
C4—C3—H3	119.6	C14—C15—H15C	109.5
C12—C4—C5	120.4 (5)	H15A—C15—H15C	109.5
C12—C4—C3	115.5 (4)	H15B—C15—H15C	109.5
C5—C4—C3	124.1 (5)	O2—C16—C17	106.4 (5)
C6—C5—C4	120.6 (5)	O2—C16—H16A	110.4
C6—C5—H5	119.7	C17—C16—H16A	110.4
C4—C5—H5	119.7	O2—C16—H16B	110.4
C5—C6—C7	121.2 (5)	C17—C16—H16B	110.4
C5—C6—H6	119.4	H16A—C16—H16B	108.6
C7—C6—H6	119.4	C18—C17—C16	119.1 (8)
C11—C7—C8	116.4 (5)	C18—C17—H17A	107.5
C11—C7—C6	119.1 (5)	C16—C17—H17A	107.5
C8—C7—C6	124.5 (5)	C18—C17—H17B	107.5
C9—C8—C7	121.6 (5)	C16—C17—H17B	107.5
C9—C8—H8	119.2	H17A—C17—H17B	107.0
C7—C8—H8	119.2	C17—C18—H18A	109.5
C8—C9—C10	118.2 (5)	C17—C18—H18B	109.5
C8—C9—H9	120.9	H18A—C18—H18B	109.5
C10—C9—H9	120.9	C17—C18—H18C	109.5
N2—C10—O2	113.0 (4)	H18A—C18—H18C	109.5
N2—C10—C9	122.0 (5)	H18B—C18—H18C	109.5
			
N2—Cd1—N1—C1	179.1 (4)	Cd1—N2—C10—O2	5.6 (6)
Cl2—Cd1—N1—C1	61.9 (4)	C11—N2—C10—C9	1.4 (7)
Cl1—Cd1—N1—C1	−82.0 (4)	Cd1—N2—C10—C9	−173.5 (4)
N2—Cd1—N1—C12	−5.3 (3)	C16—O2—C10—N2	171.6 (5)
Cl2—Cd1—N1—C12	−122.5 (3)	C16—O2—C10—C9	−9.3 (8)
Cl1—Cd1—N1—C12	93.6 (3)	C8—C9—C10—N2	−0.6 (8)
N1—Cd1—N2—C10	−179.2 (4)	C8—C9—C10—O2	−179.6 (5)
Cl2—Cd1—N2—C10	−65.1 (4)	C10—N2—C11—C7	−0.6 (7)
Cl1—Cd1—N2—C10	74.2 (4)	Cd1—N2—C11—C7	174.7 (3)
N1—Cd1—N2—C11	5.8 (3)	C10—N2—C11—C12	179.0 (4)
Cl2—Cd1—N2—C11	119.8 (3)	Cd1—N2—C11—C12	−5.7 (5)
Cl1—Cd1—N2—C11	−100.8 (3)	C8—C7—C11—N2	−0.8 (7)
C12—N1—C1—O1	−178.1 (4)	C6—C7—C11—N2	179.1 (4)
Cd1—N1—C1—O1	−2.7 (6)	C8—C7—C11—C12	179.6 (4)
C12—N1—C1—C2	−0.2 (7)	C6—C7—C11—C12	−0.5 (7)
Cd1—N1—C1—C2	175.2 (3)	C1—N1—C12—C4	0.6 (7)
C13—O1—C1—N1	−169.7 (5)	Cd1—N1—C12—C4	−175.3 (3)
C13—O1—C1—C2	12.5 (7)	C1—N1—C12—C11	−179.7 (4)
N1—C1—C2—C3	−0.8 (8)	Cd1—N1—C12—C11	4.5 (5)
O1—C1—C2—C3	176.8 (5)	C5—C4—C12—N1	−179.8 (4)
C1—C2—C3—C4	1.5 (8)	C3—C4—C12—N1	0.1 (7)
C2—C3—C4—C12	−1.1 (7)	C5—C4—C12—C11	0.5 (7)
C2—C3—C4—C5	178.7 (5)	C3—C4—C12—C11	−179.7 (4)
C12—C4—C5—C6	−1.0 (7)	N2—C11—C12—N1	0.8 (6)
C3—C4—C5—C6	179.2 (5)	C7—C11—C12—N1	−179.6 (4)
C4—C5—C6—C7	0.7 (8)	N2—C11—C12—C4	−179.4 (4)
C5—C6—C7—C11	0.0 (8)	C7—C11—C12—C4	0.2 (6)
C5—C6—C7—C8	180.0 (5)	C1—O1—C13—C14	77.9 (7)
C11—C7—C8—C9	1.5 (7)	O1—C13—C14—C15	57.3 (9)
C6—C7—C8—C9	−178.4 (5)	C10—O2—C16—C17	170.5 (6)
C7—C8—C9—C10	−0.8 (8)	O2—C16—C17—C18	55.8 (12)
C11—N2—C10—O2	−179.6 (4)		
